# The evolving burden of drug use disorders: a comprehensive epidemiological analysis from the 2021 Global Burden of Disease study

**DOI:** 10.3389/fpsyt.2025.1647269

**Published:** 2025-10-01

**Authors:** Yujuan Liu, Ning Zhang, Weifang Ren, Xiaoqun Lv, Shan Ran, Xiaofang Tan, Qiongyue Zhao

**Affiliations:** Department of Pharmacy, Jinshan Hospital Affiliated to Fudan University, Shanghai, China

**Keywords:** Global Burden of Disease, drug use disorders, socio-demographic index, opioid, health disparities

## Abstract

**Background:**

Drug use disorders (DUDs) pose a substantial global health burden, yet comprehensive analyses of their epidemiological trends, sociodemographic drivers, and cross-national disparities remain limited. Leveraging data from the Global Burden of Disease (GBD) 2021 study, we evaluated the global, regional, and national burden of DUDs from 1990 to 2021, focusing on opioids, cannabis, cocaine, amphetamines, and other substances.

**Methods:**

Using GBD 2021 data, we analyzed age-standardized incidence, prevalence, mortality, and disability-adjusted life years (DALYs) for DUDs across 204 countries. Bayesian meta-regression (DisMod-MR 2.1) and age-period-cohort modeling were applied to quantify trends, stratified by sex, age, region, and socio-demographic Index (SDI). Uncertainty intervals (UIs) were derived from 1,000 posterior draws.

**Results:**

Globally, incident DUD cases increased by 36% (95% UI: 31%–40%) from 1990 to 2021, while mortality more than doubled (122%, 95% UI: 100%–149%). Despite an 8% decline in age-standardized incidence rates (184.31 to 169.39 per 100,000), mortality rates rose by 31% (1.26 to 1.65 per 100,000), and DALYs increased by 15% (166.44 to 190.97 per 100,000). High-income North America experienced an 11.2-fold surge in mortality (6,125 to 74,451 deaths), contrasting with East Asia’s 15% reduction in incident cases. Opioid-related DALYs rose by 32% (103.69 to 137.15 per 100,000), driven by high-SDI regions, while cocaine-related mortality doubled (108%, 0.07 to 0.15 per 100,000). Males aged 20–24 had 1.35-fold higher incidence than females (386.01 vs. 285.59 per 100,000), with mortality peaking at ages 25–29 (3.45 vs. 1.12 per 100,000). SDI exhibited a strong positive correlation with DALYs (Spearman ρ=0.70, *p*<0.01), though amphetamine use disorders peaked at moderate SDI levels (0.6–0.8).

**Conclusion:**

The escalating mortality and DALYs despite declining incidence highlight systemic gaps in harm reduction and treatment access, particularly in high-SDI nations. Opioid and cocaine crises demand urgent regulatory reforms and integrated care models. Global disparities underscore the need for context-specific interventions addressing socioeconomic determinants, polysubstance use, and aging populations. Policymakers must prioritize data-driven strategies aligned with SDG 3 targets (good health and well-being) to mitigate the growing burden of DUDs.

## 1 Introduction

Drug use disorders (DUDs) are chronic, relapsing conditions characterized by the compulsive use of psychoactive substances despite significant physical, psychological, or social harm (1, 2). Clinically defined by the *Diagnostic and Statistical Manual of Mental Disorders, Fifth Edition* (DSM-5), DUDs encompass criteria such as impaired control over substance use, social impairment, risky use, and pharmacological tolerance or withdrawal (3). According to the World Health Organization (WHO), DUDs affect approximately 296 million people globally, with opioids, cannabis, and stimulants, e.g., cocaine, amphetamines being the most commonly misused substances (4). The consequences of DUDs are multifaceted, spanning individual and societal levels. Critically, unaddressed DUDs undermine progress toward United Nations Sustainable Development Goal 3 (good health and well-being for all at all ages) by perpetuating health disparities and straining public health systems (5).

In recent years, the prevalence and incidence of DUDs have risen significantly due to globalization, urbanization, and rapid socioeconomic transformations, especially in High-Income North America. Against this backdrop, disparities in healthcare resources, legal frameworks, and cultural attitudes have contributed to substantial heterogeneity in the burden of DUDs across different regions and countries (6, 7). The Global Burden of Disease (GBD) study has played a pivotal role in quantifying the worldwide impact of DUDs. According to GBD 2010, illicit drug dependence accounted for 20 million disability-adjusted life years (DALYs) globally, with opioids representing the leading contributor and high-income countries disproportionately affected (8). More recent data highlight the escalating crisis in the United States, where the DUD burden remains severe—particularly among males, young adults, and opioid users—with persistently high rates in states such as West Virginia and projections indicating sustained increases in the coming decades (9). While GBD studies have been instrumental in assessing the global burden of DUDs and advocating for evidence-based interventions, most existing research remains geographically limited, focusing on select high-income nations while neglecting broader global trends (9–12). Furthermore, methodological refinements in statistical approaches could enhance the accuracy and validity of burden estimates (13).

Given these gaps, this study leverages the latest GBD data to systematically evaluate the epidemiological trends of DUDs—including incidence, prevalence, and DALYs—from 1990 to the present. Utilizing advanced Bayesian statistical modeling, we conduct a comparative analysis of cross-national variations in DUD burden, examining how economic and healthcare system factors influence these trends. Our findings aim to inform targeted prevention and intervention strategies, addressing critical disparities in global DUD management.

## 2 Methods

### 2.1 Data sources and study design

This study utilized data from the Global Burden of Diseases, Injuries, and Risk Factors Study (GBD) 2021, accessed through the Global Health Data Exchange (GHDx) query tool, to analyze the global burden of DUDs from 1990 to 2021 (Institute for Health Metrics and Evaluation, 2024). The dataset included estimates of incidence, prevalence, mortality, and DALYs, stratified by age, sex, country, and region. The Socio-demographic Index (SDI), developed by the GBD collaboration, integrates lag-distributed income per capita, average educational attainment (ages 15+), and total fertility rate. SDI values (range: 0–1) were obtained directly from GHDx. Countries were stratified into SDI quintiles (low: <0.45; lower-middle: 0.45–0.61; middle: 0.61–0.69; high-middle: 0.69–0.81; high: >0.81), consistent with GBD 2021 protocols. Socioeconomic influences were assessed using the SDI to facilitate cross-national comparisons of development and healthcare system performance.

### 2.2 Burden estimation methods

DUDs were defined according to the DSM-5 and the International Classification of Diseases (ICD-10) (WHO, 1992), encompassing opioid, cocaine, cannabis, and amphetamine use disorders, as well as other DUDs. Burden estimates were generated using DisMod-MR 2.1, a Bayesian meta-regression tool, to ensure cross-country comparability. Age standardization was performed using the WHO standard population, with results reported as both absolute numbers and age-standardized rates per 100,000 population. Mortality estimates reflect cause-specific deaths directly attributable to SUDs (e.g., overdoses, substance-induced organ failure), as defined by GBD cause-of-death models.

### 2.3 Temporal and spatial trend analysis

Temporal trends were analyzed using the estimated annual percentage change (EAPC), calculated via regression models to quantify trends in age-standardized rates and absolute burden, and age-period-cohort (APC) analysis to decompose trends into age, period, and cohort effects. APC analysis used the intrinsic estimator to disentangle effects, with cohort defined by 5-year intervals. Countries were stratified into five SDI quintiles (low, lower-middle, middle, high-middle, and high SDI) to evaluate disparities in DUD burden across development levels, with additional stratification by GBD super-regions.

### 2.4 Statistical analysis and uncertainty quantification

Statistical uncertainty was quantified using 1000 posterior draws, with 95% uncertainty intervals (UIs) representing the 2.5th–97.5th percentile range of estimates. All analyses were conducted in R (version 4.3.3), and a two-tailed p-value <0.05 was considered statistically significant. This methodology aligns with established GBD protocols (14), ensuring robust and comparable estimates of the global burden of DUDs.

## 3 Results

### 3.1 Global trends in DUDs

Globally, the number of incident cases increased by 36% (95% UI: 31%–40%), from 10.04 million (95% UI: 8.54–11.53) in 1990 to 13.61 million (95% UI: 11.63–15.67) in 2021(see **Supplementary Table S1**). Similarly, prevalent cases rose by 34% (95% UI: 29%–40%), from 39.62 million (95% UI: 34.07–46.42) to 53.12 million (95% UI: 47.00–60.95). Mortality counts more than doubled (122%, 95% UI: 100%–149%), increasing from 61,774 (95% UI: 57,329–66,898) to 137,278 (95% UI: 129,269–146,181). DALYs counts also surged by 75% (95% UI: 65%–85%), from 8.91 million (95% UI: 7.06–10.63) to 15.56 million (95% UI: 12.75–18.12). These trends reflect both population growth and worsening health burdens.

**Figure 1:**
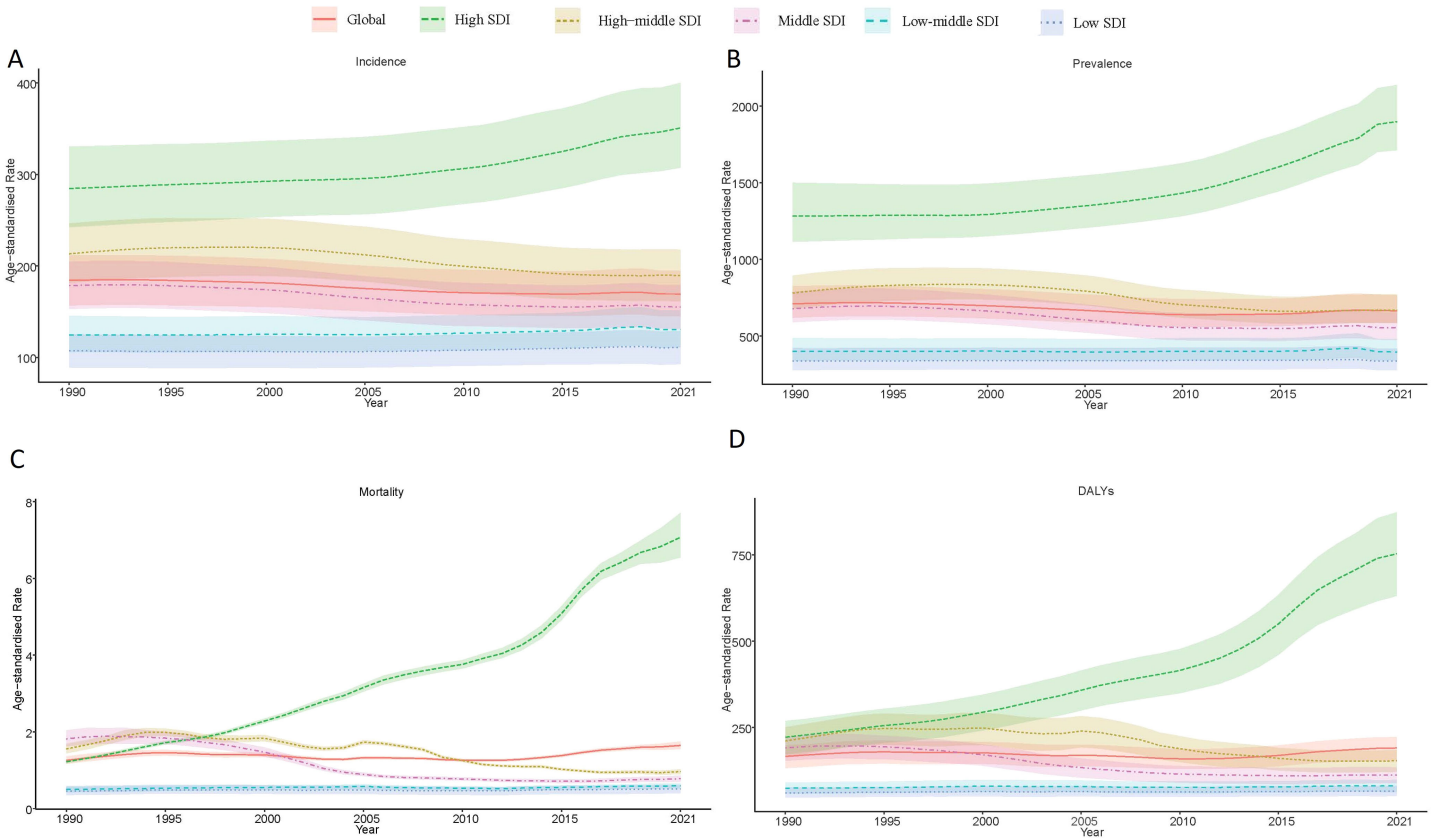
Global trends in drug use disorders from 1990–2021. **(A)** Age-standardized incidence rate of drug use disorders by SDI quintile. **(B)** Age-standardized prevalence rate of drug use disorders by SDI quintile. **(C)** Age-standardized mortality rate due to drug use disorders by SDI quintile. **(D)** Age-standardized Disability-Adjusted Life Years (DALYs) for drug use disorders by SDI quintile.

From 1990 to 2021, the global age-standardized incidence rate of DUDs declined from 184.31 (95% UI: 156.91–211.67) to 169.39 (95% UI: 145.14–195.01) per 100,000, representing an 8% decrease (**Table 1**, **Figure 1**). Similarly, prevalence rates fell by 6%, from 709.15 (95% UI: 618.81–824.54) to 663.80 (95% UI: 584.52–766.14) per 100,000. In contrast, mortality rates increased by 31%, rising from 1.26 (95% UI: 1.17–1.37) to 1.65 (95% UI: 1.55–1.75) per 100,000, while DALYs rate grew by 15%, from 166.44 (95% UI: 132.55–198.40) to 190.97 (95% UI: 156.11–222.79) per 100,000. These trends reflect a paradoxical global pattern of reduced incidence but heightened health burden.

**Table 1:** Global and regional level burden of drug use disorders stratified by incidence, prevalence, mortality and DALYs, 1990-2021.

	Incidence			Prevalence			Mortality			DALYs		
	Age-standardised rate (per 100 000 population, 95%UI)		Percentage change of rates, 1990-2021 (95%UI)	Age-standardised rate (per 100 000 population, 95%UI)		Percentage change of rates, 1990-2021 (95%UI)	Age-standardised rate (per 100 000 population, 95%UI)		Percentage change of rates, 1990-2021 (95%UI)	Age-standardised rate (per 100 000 population, 95%UI)		Percentage change of rates, 1990-2021 (95%UI)
	1990	2021		1990	2021		1990	2021		1990	2021	
Global	184.31 (156.91-211.67)	169.39 (145.14-195.01)	-0.08 (-0.10--0.06)	709.15 (618.81-824.54)	663.80 (584.52-766.14)	-0.06 (-0.10--0.03)	1.26 (1.17-1.37)	1.65 (1.55-1.75)	0.31 (0.18-0.46)	166.44 (132.55-198.40)	190.97 (156.11-222.79)	0.15 (0.09-0.21)
Regional												
Andean Latin America	144.70 (120.69-169.42)	147.25 (123.64-171.11)	0.02 (-0.02-0.06)	477.19 (404.11-573.00)	480.27 (406.37-576.63)	0.01 (-0.04-0.05)	0.36 (0.31-0.42)	0.52 (0.43-0.64)	0.44 (0.11-0.86)	83.02 (62.22-104.32)	91.59 (71.54-113.89)	0.10 (0.01-0.21)
Australasia	477.15 (415.54-555.64)	425.48 (369.38-483.04)	-0.11 (-0.19--0.03)	2231.60 (1996.93-2541.13)	1819.35 (1632.77-2054.70)	-0.18 (-0.23--0.13)	2.17 (2.03-2.33)	4.41 (3.94-4.89)	1.03 (0.77-1.31)	365.23 (294.85-430.61)	464.24 (387.42-539.76)	0.27 (0.17-0.38)
Caribbean	180.64 (149.44-218.09)	180.09 (147.21-220.40)	-0.00 (-0.04-0.04)	727.81 (576.21-930.60)	733.78 (568.30-946.83)	0.01 (-0.03-0.05)	0.21 (0.19-0.23)	0.40 (0.34-0.47)	0.90 (0.56-1.30)	90.76 (65.78-116.08)	92.30 (70.37-116.99)	0.02 (-0.04-0.09)
Central Asia	165.80 (140.28-195.03)	169.72 (143.57-197.09)	0.02 (-0.01-0.06)	549.62 (461.22-662.75)	574.49 (483.62-687.87)	0.05 (0.02-0.08)	0.51 (0.44-0.58)	0.92 (0.78-1.08)	0.82 (0.48-1.25)	135.73 (100.13-171.36)	158.77 (122.14-192.69)	0.17 (0.09-0.28)
Central Europe	175.85 (147.21-208.16)	184.24 (155.27-214.63)	0.05 (0.01-0.08)	631.15 (523.55-762.89)	662.66 (571.49-776.46)	0.05 (-0.01-0.11)	0.61 (0.56-0.67)	0.72 (0.67-0.78)	0.17 (0.05-0.30)	98.28 (77.04-120.40)	113.79 (90.95-137.08)	0.16 (0.11-0.22)
Central Latin America	141.32 (118.00-165.85)	144.04 (121.38-167.38)	0.02 (-0.00-0.05)	489.62 (422.16-578.32)	529.59 (460.90-609.70)	0.08 (0.04-0.13)	0.37 (0.35-0.38)	0.43 (0.38-0.48)	0.16 (0.01-0.32)	86.88 (65.99-109.39)	88.41 (68.83-109.78)	0.02 (-0.02-0.07)
Central Sub-Saharan Africa	107.56 (89.53-127.51)	110.05 (91.77-129.82)	0.02 (-0.02-0.07)	297.75 (238.08-387.13)	306.32 (246.24-397.68)	0.03 (0.01-0.05)	0.32 (0.19-0.49)	0.38 (0.22-0.58)	0.19 (-0.13-0.67)	52.77 (39.86-67.88)	59.27 (43.57-75.47)	0.12 (0.01-0.27)
East Asia	218.15 (184.94-253.87)	173.93 (146.09-204.63)	-0.20 (-0.24--0.16)	810.69 (697.85-954.84)	589.83 (494.67-703.92)	-0.27 (-0.32--0.22)	2.69 (2.35-3.08)	0.69 (0.57-0.83)	-0.74 (-0.80--0.67)	268.49 (217.53-316.18)	117.23 (89.99-144.50)	-0.56 (-0.62--0.52)
Eastern Europe	262.36 (224.86-300.18)	275.72 (238.80-312.90)	0.05 (0.01-0.09)	963.68 (829.48-1114.46)	1041.24 (908.44-1198.50)	0.08 (0.04-0.12)	2.38 (2.22-2.57)	3.41 (3.11-3.73)	0.43 (0.26-0.64)	323.60 (257.79-382.26)	403.12 (337.84-468.51)	0.25 (0.16-0.35)
Eastern Sub-Saharan Africa	98.94 (81.32-117.53)	101.09 (83.77-119.60)	0.02 (-0.01-0.05)	327.74 (260.28-419.69)	325.17 (257.73-422.17)	-0.01 (-0.04-0.01)	0.60 (0.40-0.86)	0.67 (0.46-0.86)	0.11 (-0.12-0.37)	60.83 (47.07-78.07)	66.58 (51.76-82.03)	0.09 (-0.02-0.20)
High-income Asia Pacific	208.76 (170.85-253.22)	204.38 (168.19-247.27)	-0.02 (-0.05-0.01)	798.76 (660.32-1022.92)	781.29 (644.13-995.33)	-0.02 (-0.04-0.01)	0.11 (0.10-0.11)	0.17 (0.16-0.18)	0.62 (0.51-0.75)	89.87 (63.71-119.15)	90.07 (65.11-117.63)	0.00 (-0.03-0.04)
High-income North America	366.58 (312.82-426.29)	520.07 (454.13-592.82)	0.42 (0.33-0.52)	1997.89 (1722.10-2324.30)	3668.01 (3323.49-4067.36)	0.84 (0.67-1.03)	1.93 (1.83-2.04)	18.42 (16.81-20.33)	8.55 (7.54-9.84)	352.05 (270.73-427.06)	1836.34 (1547.74-2122.45)	4.22 (3.72-4.83)
North Africa and Middle East	134.12 (112.41-158.09)	143.52 (120.87-169.07)	0.07 (0.05-0.10)	378.78 (326.81-439.00)	422.68 (369.99-485.49)	0.12 (0.08-0.16)	1.20 (1.05-1.40)	1.24 (1.10-1.42)	0.04 (-0.15-0.21)	148.73 (117.32-181.45)	161.95 (129.92-193.35)	0.09 (0.01-0.17)
Oceania	172.19 (139.24-211.99)	173.25 (141.60-212.00)	0.01 (-0.03-0.04)	668.94 (495.56-902.66)	672.72 (503.93-893.96)	0.01 (-0.01-0.03)	0.23 (0.15-0.33)	0.16 (0.12-0.22)	-0.30 (-0.46--0.08)	71.33 (51.57-93.20)	68.81 (49.71-89.73)	-0.04 (-0.11-0.04)
South Asia	120.99 (100.92-142.05)	131.41 (109.78-153.28)	0.09 (0.05-0.12)	380.92 (311.84-479.25)	391.33 (327.33-483.68)	0.03 (-0.02-0.08)	0.59 (0.50-0.67)	0.64 (0.56-0.72)	0.10 (-0.09-0.30)	71.11 (56.10-87.26)	78.68 (62.13-95.44)	0.11 (0.03-0.18)
Southeast Asia	137.34 (113.22-161.97)	141.48 (116.93-166.01)	0.03 (0.01-0.05)	519.61 (419.67-644.87)	524.30 (424.34-649.16)	0.01 (-0.02-0.04)	0.28 (0.24-0.33)	0.34 (0.29-0.42)	0.23 (-0.03-0.56)	68.29 (50.24-88.66)	71.21 (52.71-91.13)	0.04 (-0.02-0.11)
Southern Latin America	185.95 (156.40-219.07)	196.13 (167.54-227.34)	0.05 (0.00-0.11)	729.13 (636.60-843.34)	815.88 (730.49-926.72)	0.12 (0.05-0.19)	0.07 (0.07-0.08)	0.20 (0.18-0.23)	1.71 (1.35-2.16)	104.52 (72.10-139.02)	110.29 (79.08-143.92)	0.06 (-0.02-0.15)
Southern Sub-Saharan Africa	161.58 (139.28-185.37)	161.51 (137.31-186.47)	-0.00 (-0.03-0.04)	670.31 (586.96-776.34)	639.35 (539.97-771.88)	-0.05 (-0.09-0.01)	1.31 (1.10-1.46)	1.32 (1.20-1.45)	0.01 (-0.12-0.21)	175.43 (138.47-210.37)	143.29 (117.00-169.05)	-0.18 (-0.23--0.12)
Tropical Latin America	174.68 (146.99-208.86)	180.40 (153.43-207.80)	0.03 (-0.03-0.09)	903.62 (741.68-1122.15)	888.21 (750.00-1066.61)	-0.02 (-0.09-0.05)	0.09 (0.09-0.10)	0.57 (0.54-0.62)	5.24 (4.76-5.73)	102.09 (71.29-133.35)	129.66 (98.32-160.11)	0.27 (0.19-0.40)
Western Europe	290.50 (248.87-336.73)	302.00 (262.87-348.16)	0.04 (-0.01-0.09)	1126.22 (986.69-1287.06)	1201.17 (1081.17-1351.18)	0.07 (0.02-0.11)	1.32 (1.28-1.36)	2.28 (2.19-2.37)	0.73 (0.64-0.82)	211.34 (170.89-250.97)	276.35 (230.25-322.25)	0.31 (0.27-0.35)
Western Sub-Saharan Africa	88.51 (72.58-106.10)	94.68 (79.60-111.36)	0.07 (0.03-0.12)	234.19 (195.34-284.58)	236.64 (199.54-285.92)	0.01 (-0.02-0.04)	0.07 (0.05-0.10)	0.05 (0.04-0.07)	-0.27 (-0.40--0.07)	37.90 (27.48-48.72)	37.27 (26.73-48.09)	-0.02 (-0.05-0.02)
SDI index												
High SDI	284.65 (242.42-330.56)	350.90 (307.36-400.20)	0.23 (0.18-0.29)	1282.80 (1114.26-1499.92)	1897.69 (1710.93-2137.33)	0.48 (0.39-0.59)	1.22 (1.18-1.26)	7.07 (6.54-7.71)	4.79 (4.31-5.42)	222.34 (173.15-269.45)	752.61 (630.61-872.87)	2.38 (2.11-2.73)
High-middle SDI	213.21 (181.57-246.44)	189.65 (161.53-218.09)	-0.11 (-0.13--0.09)	779.94 (678.13-894.16)	667.46 (584.48-771.03)	-0.14 (-0.17--0.12)	1.56 (1.44-1.69)	0.97 (0.90-1.03)	-0.38 (-0.46--0.31)	211.32 (166.86-251.33)	153.92 (122.22-185.21)	-0.27 (-0.31--0.24)
Middle SDI	178.51 (152.88-204.74)	155.19 (131.25-179.27)	-0.13 (-0.16--0.10)	677.71 (589.07-788.40)	552.53 (475.41-653.60)	-0.18 (-0.23--0.14)	1.82 (1.62-2.04)	0.78 (0.69-0.86)	-0.57 (-0.64--0.49)	191.36 (154.29-223.58)	112.90 (89.50-135.09)	-0.41 (-0.47--0.36)
Low-middle SDI	124.75 (105.84-145.45)	130.55 (110.96-151.41)	0.05 (0.02-0.07)	398.90 (337.37-486.57)	395.86 (339.37-479.11)	-0.01 (-0.03-0.02)	0.50 (0.43-0.58)	0.59 (0.53-0.66)	0.18 (0.02-0.36)	74.83 (59.16-91.85)	81.28 (63.74-98.29)	0.09 (0.03-0.14)
Low SDI	107.37 (88.96-125.35)	110.82 (92.59-128.79)	0.03 (0.01-0.06)	336.54 (276.97-420.79)	335.76 (278.02-416.83)	-0.00 (-0.02-0.02)	0.45 (0.35-0.59)	0.51 (0.40-0.62)	0.13 (-0.03-0.31)	61.21 (47.94-76.72)	66.47 (51.71-81.05)	0.09 (0.03-0.15)
Drug Use Disorders												
Amphetamine use disorders	22.70 (15.92-31.75)	13.72 (9.70-19.07)	-0.40 (-0.43--0.36)	186.84 (136.71-248.87)	115.99 (84.63-153.55)	-0.38 (-0.41--0.35)	0.09 (0.08-0.11)	0.12 (0.11-0.13)	0.28 (0.00-0.58)	29.63 (19.51-43.52)	20.98 (14.56-29.33)	-0.29 (-0.34--0.22)
Cannabis use disorders	48.46 (36.39-63.37)	46.77 (35.25-61.17)	-0.03 (-0.06--0.01)	298.72 (230.75-395.78)	286.23 (222.58-384.31)	-0.04 (-0.06--0.02)	_	_	_	8.63 (5.10-13.25)	8.27 (4.90-12.86)	-0.04 (-0.07--0.02)
Cocaine use disorders	3.09 (2.13-4.39)	2.87 (2.06-3.93)	-0.07 (-0.14-0.01)	54.64 (41.12-72.61)	50.63 (39.74-63.79)	-0.07 (-0.15-0.02)	0.07 (0.06-0.09)	0.15 (0.14-0.17)	1.08 (0.72-1.59)	10.91 (7.86-14.84)	13.88 (11.18-17.52)	0.27 (0.13-0.45)
Opioid use disorders	23.37 (19.58-28.48)	24.54 (20.74-29.48)	0.05 (0.02-0.09)	154.59 (131.06-181.26)	198.49 (173.42-227.22)	0.28 (0.23-0.35)	0.86 (0.76-0.93)	1.19 (1.12-1.29)	0.39 (0.27-0.54)	103.69 (81.83-122.75)	137.15 (112.29-161.39)	0.32 (0.26-0.40)
Other drug use disorders	86.69 (65.72-111.42)	81.49 (62.45-103.64)	-0.06 (-0.09--0.03)	18.65 (14.97-22.95)	18.17 (14.82-22.12)	-0.03 (-0.07-0.01)	0.24 (0.19-0.33)	0.18 (0.17-0.20)	-0.22 (-0.44--0.00)	13.58 (11.05-18.14)	10.69 (9.74-11.80)	-0.21 (-0.41--0.03)

"_", data are not available.

### 3.2 Regional variations in case counts and growth rates

Regionally, High-income North America experienced the most dramatic increases from 1990 to 2021, with mortality counts rising by 11.2-fold (95% UI: 9.8–12.9), from 6,125 (95% UI: 5,798–6,478) to 74,451 (95% UI: 67,591–82,622). Eastern Sub-Saharan Africa saw incident cases grow by 149% (95% UI: 142–157), from 167,665 (95% UI: 135,071–206,477) to 417,962 (95% UI: 340,922–513,396), reflecting demographic shifts. In contrast, East Asia saw reduced incident cases by 15% (95% UI: 9–22%), from 3.01 million (95% UI: 2.55–3.49) in 1990 to 2.55 million (95% UI: 2.13–3.03) in 2021(**Table 1**; **Supplementary Table S1**).

**Figure 2:**
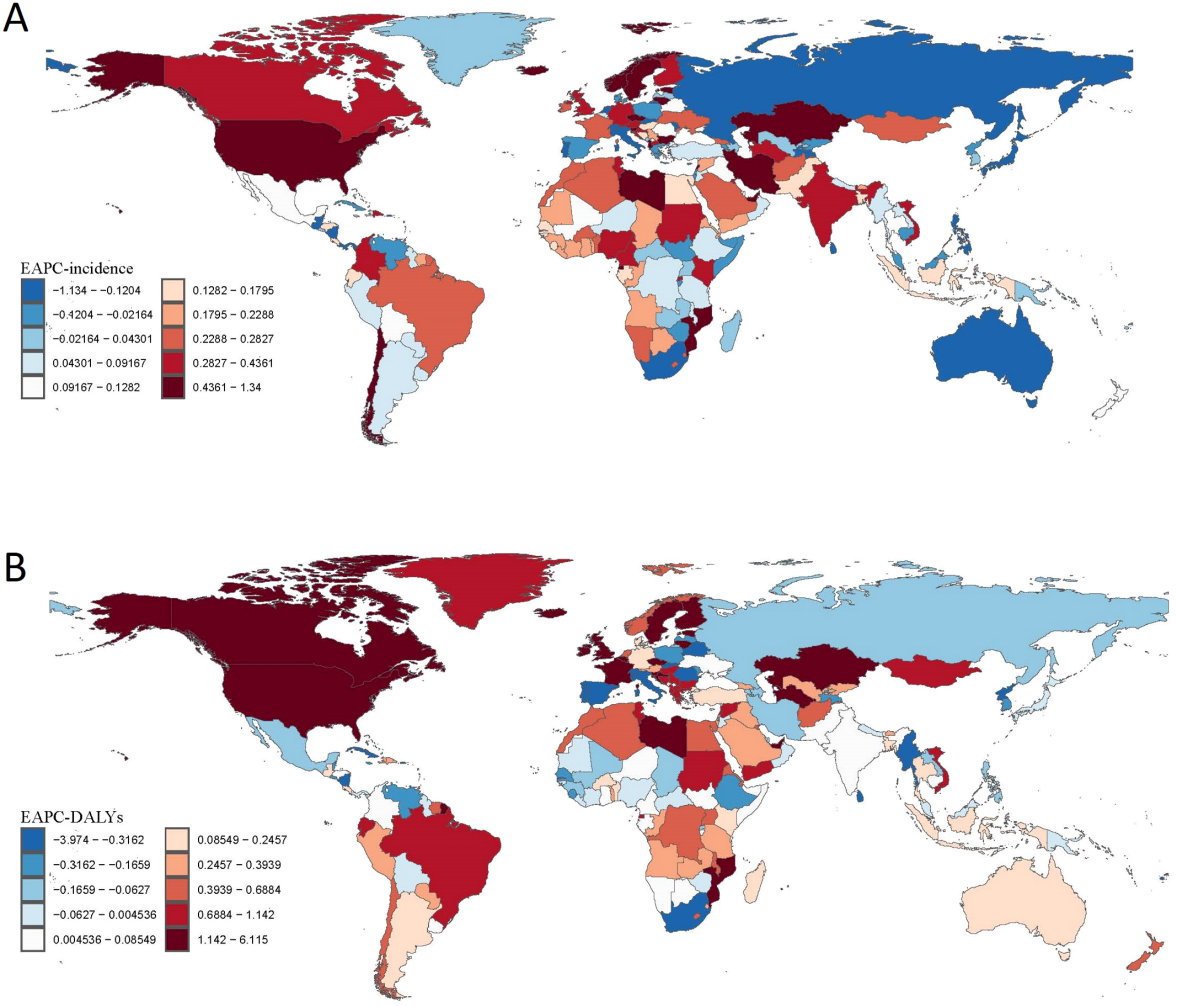
Global Spatial Distribution of Estimated Annual Percentage Change (EAPC) in Drug Use Disorders (DUD) Burden, 1990–2021. **(A)** EAPC in age-standardized incidence rates of DUD across regions and countries. **(B)** EAPC in Disability-Adjusted Life Years (DALYs) of DUD across regions and countries.

Stratified by SDI, high-SDI regions experienced the most dramatic deterioration, with mortality rates increasing 4.79-fold (reaching 7.07 per 100,000 [95% UI: 6.54-7.71]) and DALYs more than doubling (2.38-fold increase to 752.61 [95% UI: 630.61-872.87]). In contrast, middle-SDI regions demonstrated significant improvements, achieving a 41% reduction in DALYs (from 191.36 [95% UI: 154.29-223.58] to 112.90 [95% UI: 89.50-135.09]). Low-SDI regions showed minimal progress, with modest increases in both mortality (13% rise from 0.45 [95% UI: 0.35-0.59] to 0.51 [95% UI: 0.40-0.62]) and DALYs (9% increase from 61.21 [95% UI: 47.94-76.72] to 66.47 [95% UI: 51.71-81.05]) (**Figure 2**).

### 3.3 National patterns and high-burden countries

Our analysis also revealed substantial cross-national variations in DUDs burden. In 2021, the United States recorded the highest age-standardized incidence (531.19 per 100,000; 95% UI: 462.11-605.02) and prevalence rates (3,821.43 per 100,000; 95% UI: 3,450.13-4,257.62). Other high-income nations, including Australia and Canada, similarly demonstrated elevated incidence rates. Eastern European countries such as Estonia showed a high incidence coupled with relatively low mortality. China maintained notably low incidence and prevalence rates, consistent with its strict drug control policies. Iceland presented a high incidence (318.52; 95% UI: 272.44-368.13) alongside minimal mortality (0.13; 95% UI: 0.09-0.17) (**Supplementary Table S2**).

In low- and middle-income countries, distinct patterns emerged. South Africa exhibited moderate incidence (170.56 per 100,000; 95% UI: 144.68-197.27) but low mortality (1.49; 95% UI: 1.34-1.65). India reported a relatively low incidence (133.13; 95% UI: 111.12-155.54) but considerable DALYs (81.52; 95% UI: 64.47-98.94). Southeast Asian nations demonstrated intermediate burden levels, with Thailand showing an incidence of 165.55 (95% UI: 138.14-193.87) and Vietnam 173.11 (95% UI: 143.44-203.38), accompanied by DALYs of 103.34 and 140.56, respectively (**Supplementary Table S2**).

### 3.4 Global burden of specific DUDs (1990–2021)

#### 3.4.1 Amphetamine use disorders

Globally, the age-standardized incidence rate of amphetamine use disorders declined significantly by 40% (95% UI: 36%–43%), from 22.70 (95% UI: 15.92–31.75) per 100,000 in 1990 to 13.72 (95% UI: 9.70–19.07) in 2021 (see **Supplementary Figure S2**). Similarly, prevalence rates decreased by 38% (95% UI: 41–35%), from 186.84 (95% UI: 136.71–248.87) to 115.99 (95% UI: 84.63–153.55). Mortality rates, however, increased by 28% (95% UI: 0–58%), from 0.09 (95% UI: 0.08–0.11) to 0.12 (95% UI: 0.11–0.13), while DALYs decreased by 29% (95% UI: 22%–34%), from 29.63 (95% UI: 19.51–43.52) to 20.98 (95% UI: 14.56–29.33).

#### 3.4.2 Cannabis use disorders

Cannabis use disorders showed modest declines in incidence (-3%, 95% UI: -6% to-1%) and prevalence (-4%, 95% UI: -2% to -6%) (see **Supplementary Figure S3**). The incidence rate decreased from 48.46 (95% UI: 36.39–63.37) to 46.77 (95% UI: 35.25–61.17) per 100,000, while prevalence fell from 298.72 (95% UI: 230.75–395.78) to 286.23 (95% UI: 222.58–384.31). DALYs also decreased slightly (-4%, 95% UI: -7% to -2%), from 8.63 (95% UI: 5.10–13.25) to 8.27 (95% UI: 4.90–12.86). Mortality data were unavailable.

#### 3.4.3 Cocaine use disorders

Cocaine use disorders exhibited a minor decline in incidence (-7%, 95% UI: -14% to-1%), from 3.09 (95% UI: 2.13–4.39) to 2.87 (95% UI: 2.06–3.93) per 100,000, and prevalence (-7%, 95% UI: -15% to-2%), from 54.64 (95% UI: 41.12–72.61) to 50.63 (95% UI: 39.74–63.79) (see **Supplementary Figure S1**). However, mortality rates surged by 108% (95% UI: 72%–159%), from 0.07 (95% UI: 0.06–0.09) to 0.15 (95% UI: 0.14–0.17), and DALYs increased by 27% (95% UI: 13%–45%), from 10.91 (95% UI: 7.86–14.84) to 13.88 (95% UI: 11.18–17.52) (see **Supplementary Figure S4**).

#### 3.4.4 Opioid use disorders

Opioid use disorders increased in incidence (5%, 95% UI: 2%–9%), from 23.37 (95% UI: 19.58–28.48) to 24.54 (95% UI: 20.74–29.48) per 100,000, and prevalence (28%, 95% UI: 23–35%), from 154.59 (95% UI: 131.06–181.26) to 198.49 (95% UI: 173.42–227.22) (see **Supplementary Figure S1**). Mortality rose by 39% (95% UI: 27–54%), from 0.86 (95% UI: 0.76–0.93) to 1.19 (95% UI: 1.12–1.29), and DALYs increased by 32% (95% UI: 26%–40%), from 103.69 (95% UI: 81.83–122.75) to 137.15 (95% UI: 112.29–161.39) (see **Supplementary Figure S5**).

#### 3.4.5 Other DUDs

Other DUDs saw declines in incidence (−6%, 95% UI: −9% to –3%), from 86.69 (95% UI: 65.72–111.42) to 81.49 (95% UI: 62.45–103.64), and prevalence (−3%, 95% UI: –7% to –1%), from 18.65 (95% UI: 14.97–22.95) to 18.17 (95% UI: 14.82–22.12) (see **Supplementary Figure S1**). Mortality decreased by 22% (95% UI: 44–0%), from 0.24 (95% UI: 0.19–0.33) to 0.18 (95% UI: 0.17–0.20), while DALYs fell by 21% (95% UI: 41–3%), from 13.58 (95% UI: 11.05–18.14) to 10.69 (95% UI: 9.74–11.80) (see **Supplementary Figure S6**).

### 3.5 Sex and age-specific burden of DUDs

**Figure 3:**
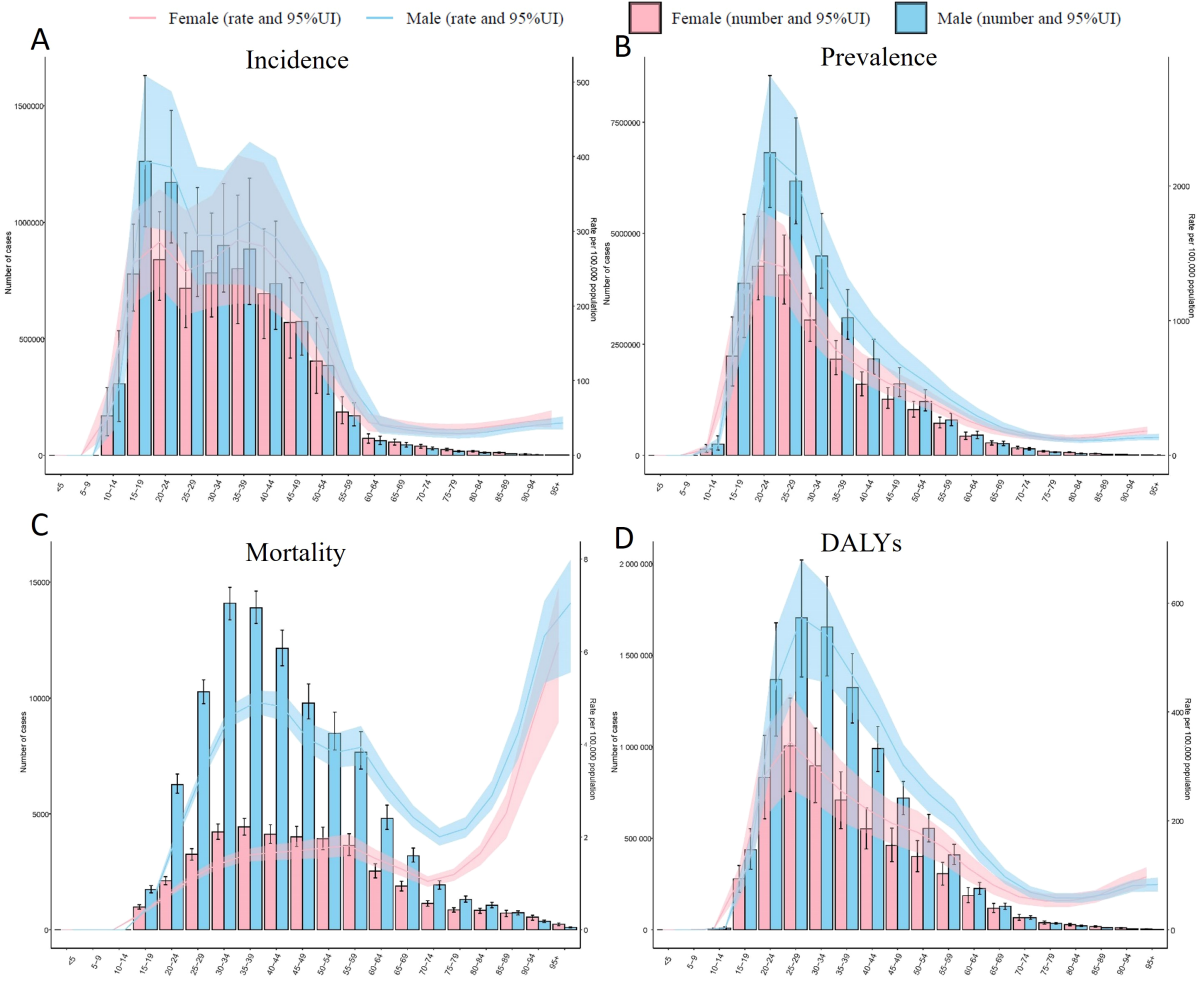
Age-specific burden of Drug Use Disorders (DUD) by sex, 1990–2021. **(A)** Age-standardized incidence rate (per 100,000) of drug use disorder (DUD) cases, stratified by sex, with 95% uncertainty intervals (UI). **(B)** Age-standardized prevalence rate (per 100,000) of DUD cases, stratified by sex, with 95% UI. **(C)** Age-standardized mortality rate (per 100,000) of DUD cases, stratified by sex, with 95% UI. **(D)** Age-standardized DALYs of DUD cases, stratified by sex, with 95% UI.

The analysis revealed pronounced sex disparities across all metrics. Males consistently exhibited higher rates than females, particularly in young adulthood. For instance, among those aged 20–24 years, males had an incidence rate of 386.01 per 100,000 (95% UI: 300.36–487.59), 1.35-fold higher than females (285.59; 95% UI: 226.18–356.01) (**Figure 3**). Mortality disparities were starkest in the 25–29 age group, with male rates (3.45; 95% UI: 3.28–3.63) triple those of females (1.12; 95% UI: 1.03–1.20). The burden peaked in early adulthood: males aged 35–39 years showed the highest incidence (312.74; 95% UI: 228.33–419.79), while females aged 30–34 years had the highest incidence (261.94; 95% UI: 198.33–347.49). Mortality surged 6.4-fold in males between ages 15–19 (0.54; 95% UI: 0.50–0.60) and 25–29 (3.45), underscoring escalating risks in early adulthood.

The burden declined markedly after age 60 but remained non-negligible. For example, males aged 60–64 had an incidence of 40.28 per 100,000 (95% UI: 29.39–53.03), 87% lower than their peak, yet DALYs persisted at 144.75 (95% UI: 123.94–165.66) (**Figure 3**). Notably, sex differences narrowed in older age groups: females ≥95 years had mortality rates (6.19; 95% UI: 4.48–7.39) approaching males (7.05; 95% UI: 5.55–7.98), suggesting cumulative health risks in aging populations. Critical data gaps included null values for individuals <15 years (e.g., <5 years: 0/100,000), likely reflecting underreporting or negligible incidence. Wide uncertainty intervals, such as for male adolescents aged 10–14 (incidence: 89.28; 95% UI: 42.03–155.51), highlight the need for enhanced surveillance.

### 3.6 The relationship between SDI and DUDs

**Figure 4:**
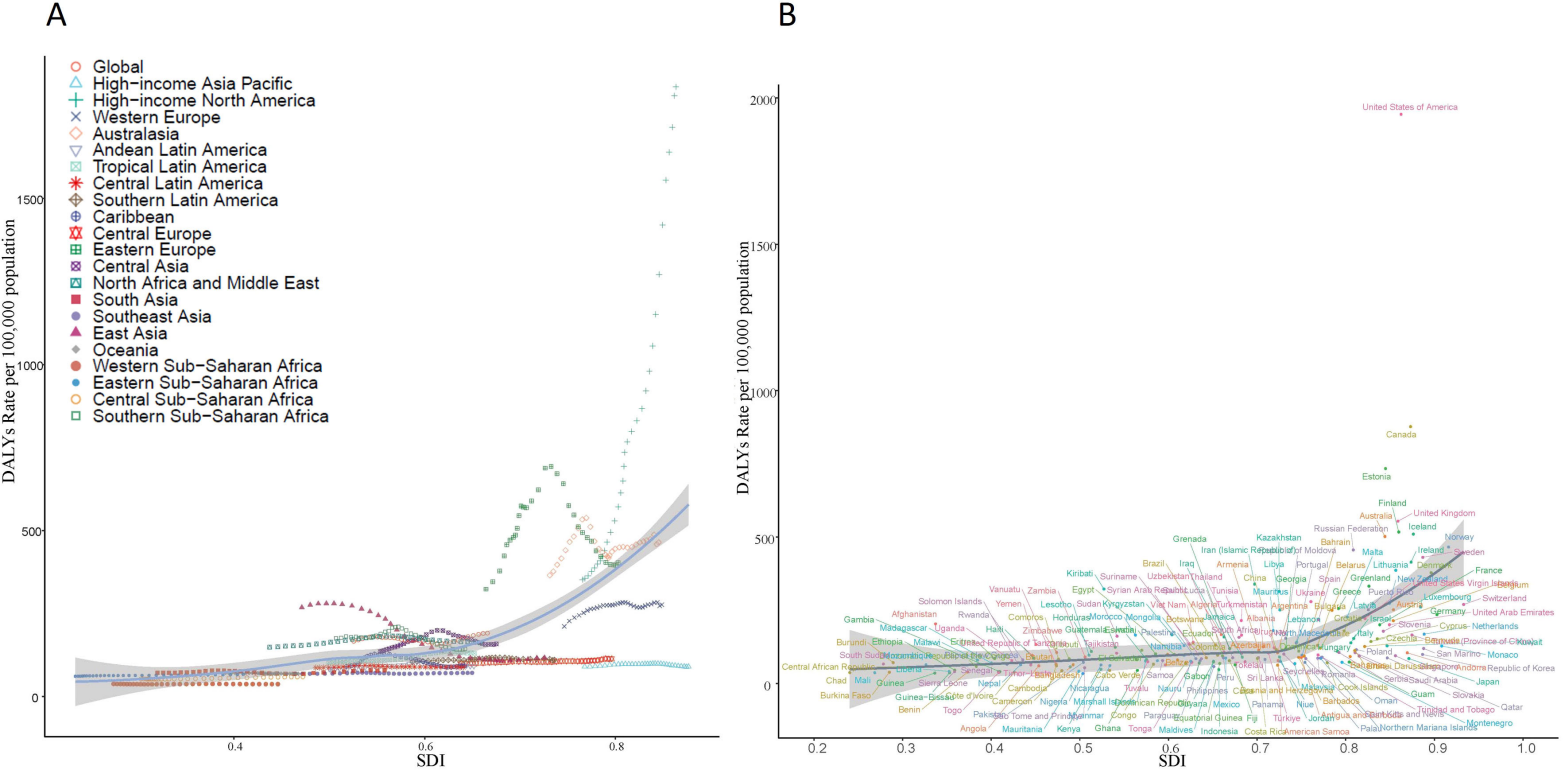
The relationship between age-standardized rate and sociodemographic index (SDI) of Drug Use Disorders (DUD) in 2021. **(A)** The relationship between SDI and age-standardized DALYs in 21 GBD regions in 2021. **(B)** The relationship between SDI and age-standardized DALYs in 204 countries in 2021.

A significant positive correlation was observed between the SDI and the incidence, prevalence, mortality, and DALYs rates of DUD. Specifically, regions with higher SDI values (e.g., North America, Western Europe, and Australasia) exhibited a greater burden of substance use disorders, whereas regions with lower SDI values (e.g., sub-Saharan Africa, South Asia, and parts of the Middle East) demonstrated a lower burden (**Figure 4**). Spearman correlation analysis revealed a strong positive association between SDI and DALYs rates for substance use disorders (p= 0.70, p < 0.01). This trend remained consistent across different regions and populations, although its magnitude varied depending on socioeconomic, cultural, and policy contexts. Furthermore, similar patterns were observed for specific types of substance use disorders, with higher SDI regions generally reporting elevated DALYs rates compared to lower SDI regions.

However, it is noteworthy that the prevalence and incidence of amphetamine use disorders exhibited a non-linear relationship with SDI: while rates increased with rising SDI, they declined in regions with the highest SDI values (>0.8). The peak burden occurred at moderate-to-high SDI levels (0.6–0.8), with the lowest rates observed in both low-SDI regions and those with SDI > 0.8. Additionally, certain high-SDI countries (e.g., the United States) and some middle-SDI regions (e.g., parts of Eastern Europe) continued to report a disproportionately high burden of substance use disorders (**Figure 4**).

## 4 Discussion

Our study reveals a paradoxical phenomenon in the global burden of DUDs. Despite an 8% reduction in ASIR, total mortality rate more than doubled. This disparity was most extreme in high-SDI regions, where North America recorded an 11.2-fold rise in drug-related deaths. This finding suggests that while preventive measures may have reduced new cases, critical gaps remain in harm reduction and treatment interventions, leading to escalating mortality (6, 12, 15). The global decline in incidence likely reflects partial success of prevention strategies, including enhanced prescription drug regulations and educational interventions in some regions. However, the concurrent rise in mortality and DALYs indicates systemic failures in emergency care, harm reduction, and treatment retention for existing cases. This divergence underscores that prevention alone cannot address the multifaceted health risks of DUDs, necessitating comprehensive approaches integrating prevention, intervention, and long-term management (16).

In the past decades, the global architecture of drug policy underwent marked heterogeneity. High-income jurisdictions progressively supplanted punitive paradigms with harm-reduction frameworks rooted in public-health principles (17). Yet implementation remains uneven: Western Europe exhibits robust coverage, whereas Central and Eastern Europe and the Western Balkans report persistent legal and socio-cultural barriers that attenuate programme reach (18) (19). Conversely, North America experienced pharmaceutical deregulation during the same interval, precipitating unprecedented increases in prescription-opioid availability (20). This regulatory trajectory aligns with the region’s concomitant rise in age-standardised DALYs and incidence rates attributable to opioid use disorders, the highest globally recorded. In East Asia, stringent prohibitionist regimes—most notably China’s 2008 Anti-Drug Law—have coincided with declining ASR of drug-use disorders (21). Collectively, these divergent policy regimes and their differential enforcement offer a compelling explanation for the pronounced regional disparities in drug-related health burdens observed over the study period.

Notably, our study provides comprehensive epidemiological evidence establishing opioids as a predominant contributor to the global burden of DUDs. The marked rise in opioid-attributable DALYs reflects two concurrent phenomena: 1) the extensive proliferation of opioids in both medical and illicit markets, and 2) a public health crisis in high-income nations driven by overprescription, aggressive pharmaceutical marketing, and regulatory failures (22, 23). These findings suggest that despite greater healthcare resources, high-income countries face significant challenges in opioid stewardship, with inadequate distribution systems and regulatory oversight potentially exacerbating socioeconomic burdens. This evidence complements existing research on postoperative opioid risks (24) and reinforces the critical importance of robust regulatory frameworks in preventing opioid misuse, as previously emphasized by Hall and Degenhardt (25). Furthermore, our results challenge the efficacy of single-substance intervention strategies, particularly given the 108% increase in cocaine-related mortality observed in parallel with rising opioid burdens. The frequent co-use of cocaine and opioids—with their potential synergistic toxicity—presents unique clinical and public health challenges (26), underscoring the need for integrated approaches to address polysubstance use disorders (27).

DUDs are strongly associated with socioeconomic determinants including economic inequality, unemployment, low education, and weak social support systems (26, 28), with these disparities further compounded by sociocultural factors such as ethnicity, gender, and migrant status (29, 30). Empirical evidence consistently shows that regions experiencing greater economic distress exhibit elevated DUDs prevalence, with these burdens extending beyond health outcomes to impact social stability and national security (31). Particularly concerning are institutional settings such as prisons, where systemic deficiencies and social marginalization contribute to disproportionately high rates of DUDs and comorbid mental health conditions (32). Addressing these challenges requires both a deeper understanding of the complex socioeconomic-health relationships and methodological advancements in research approaches. Recent developments in disparity analysis frameworks and cost-effectiveness threshold methodologies offer improved tools for evaluating public health interventions (33, 34). Furthermore, multinational comparative studies have enhanced our capacity to quantify and address regional health inequalities (Mokdad, 2018), providing critical evidence to inform more targeted and effective policy responses to the DUDs epidemic (28).

Despite significant advances in burden assessment and intervention strategies for DUDs, several key limitations persist in current research. First, DALYs are limited by inconsistent data quality and reporting, especially in low- and middle-income countries (LMICs) where underreporting and bias are common (35). This, combined with scarce primary data, leads to reliance on models that may reduce estimate accuracy (36, 37). Second, research often focuses on single substances, ignoring polysubstance interactions and cumulative effects (38). Third, few multinational studies limit the understanding of cross-cultural DUDs variations. Additionally, underestimating mortality linked to some substances (e.g., cannabis) can result in burden miscalculations (39). Lastly, while descriptive analyses are common, rigorous evaluations of prevention and treatment interventions are lacking, hindering evidence-based policy and program development (40).

## 5 Conclusion

DUDs represent a growing global health challenge requiring multifaceted solutions. Our comprehensive analysis provides policymakers and practitioners with an evidence-based framework for developing targeted prevention and treatment strategies. Future efforts must address current data gaps while implementing integrated approaches that account for the complex interplay of clinical, social, and economic factors driving DUD burden worldwide.

## Data Availability

Publicly available datasets were analyzed in this study. This data can be found here: https://ghdx.healthdata.org/. Further inquiries can be directed to the corresponding author/s.

## Supplementary material

Supplementary Table 1 Prevalent, incident cases and disability-adjusted life years (DALYs) counts of drug use disorders (DUDs), from 1990 to 2021.

Supplementary Table 2 National patterns of drug use disorders (DUDs) in 204 countries in 2021.

Supplementary Figure 1 Global trends of age-standardized prevalence, incidence and disability-adjusted life years (DALYs) of drug use disorders (DUDs), from 1990 to 2021.

Supplementary Figure 2 Temporal trends and estimated annual percentage change (EAPC) of age-standardized incidence rate (ASIR) of Amphetamine use disorders, from 1990 to 2021.

Supplementary Figure 3 Temporal trends and estimated annual percentage change (EAPC) of age-standardized incidence rate (ASIR) of Cannabis use disorders, from 1990 to 2021.

Supplementary Figure 4 Temporal trends and estimated annual percentage change (EAPC) of disability-adjusted life years (DALYs) of Cocaine use disorders, from 1990 to 2021.

Supplementary Figure 5 Temporal trends and estimated annual percentage change (EAPC) of disability-adjusted life years (DALYs) of Opioid use disorders, from 1990 to 2021.

Supplementary Figure 6 Temporal trends and estimated annual percentage change (EAPC) of disability-adjusted life years (DALYs) of other use disorders, from 1990 to 2021.
