# Supplementary figures and images for: The evolving burden of drug use disorders: a comprehensive epidemiological analysis from the 2021 Global Burden of Disease study
Source: Front Psychiatry. 2025 Oct 1;16:1647269. doi: 10.3389/fpsyt.2025.1647269 (PMC12522400; doi:10.3389/fpsyt.2025.1647269)

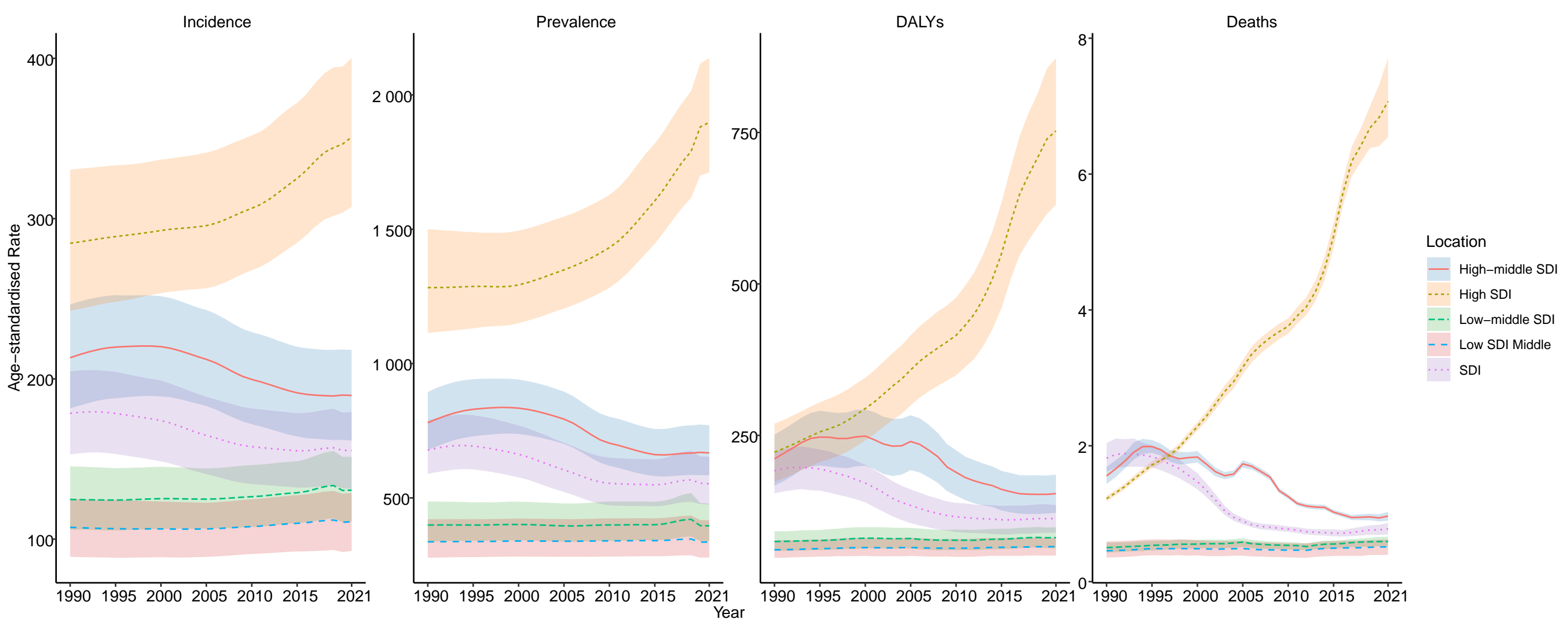

Supplement: Supplementary Figure 1 — Global trends of age-standardized prevalence, incidence and disability-adjusted life years (DALYs) of drug use disorders (DUDs), from 1990 to 2021. [file DataSheet1.pdf]

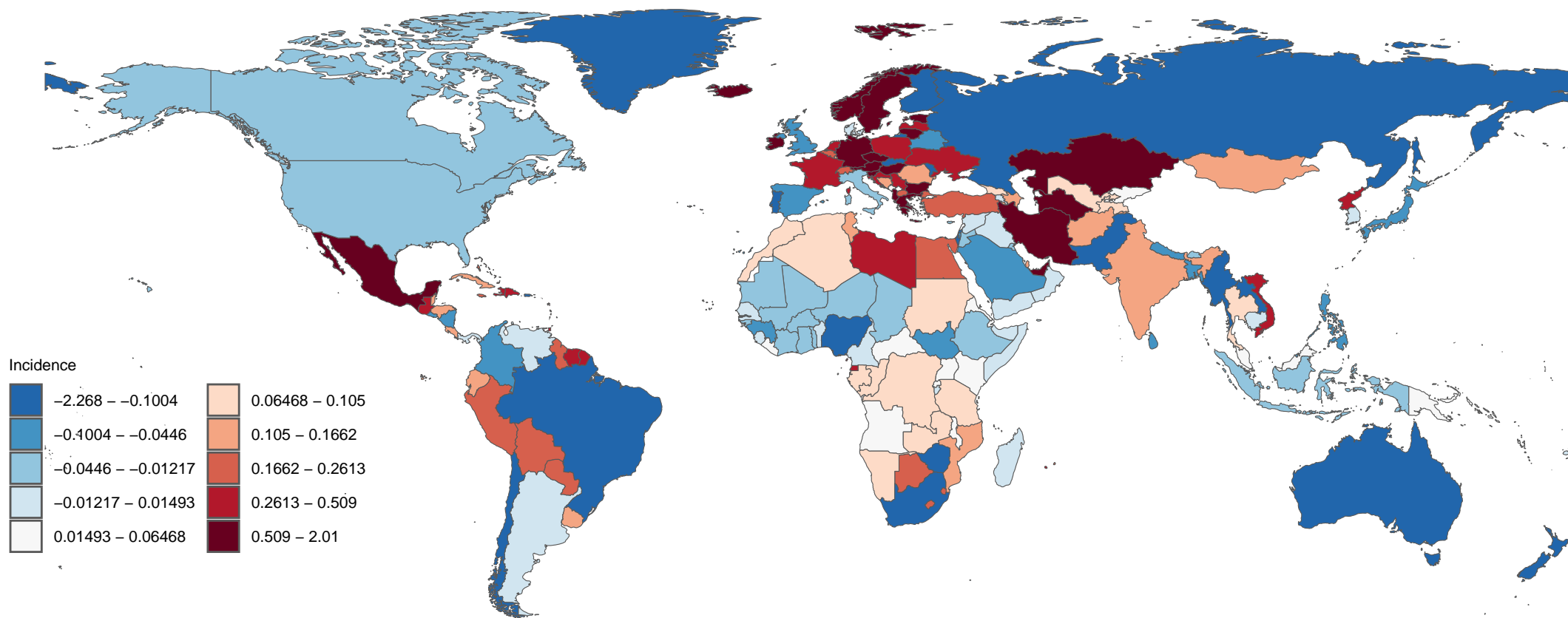

Supplement: Supplementary Figure 2 — Temporal trends and estimated annual percentage change (EAPC) of age-standardized incidence rate (ASIR) of Amphetamine use disorders, from 1990 to 2021. [file DataSheet2.pdf]

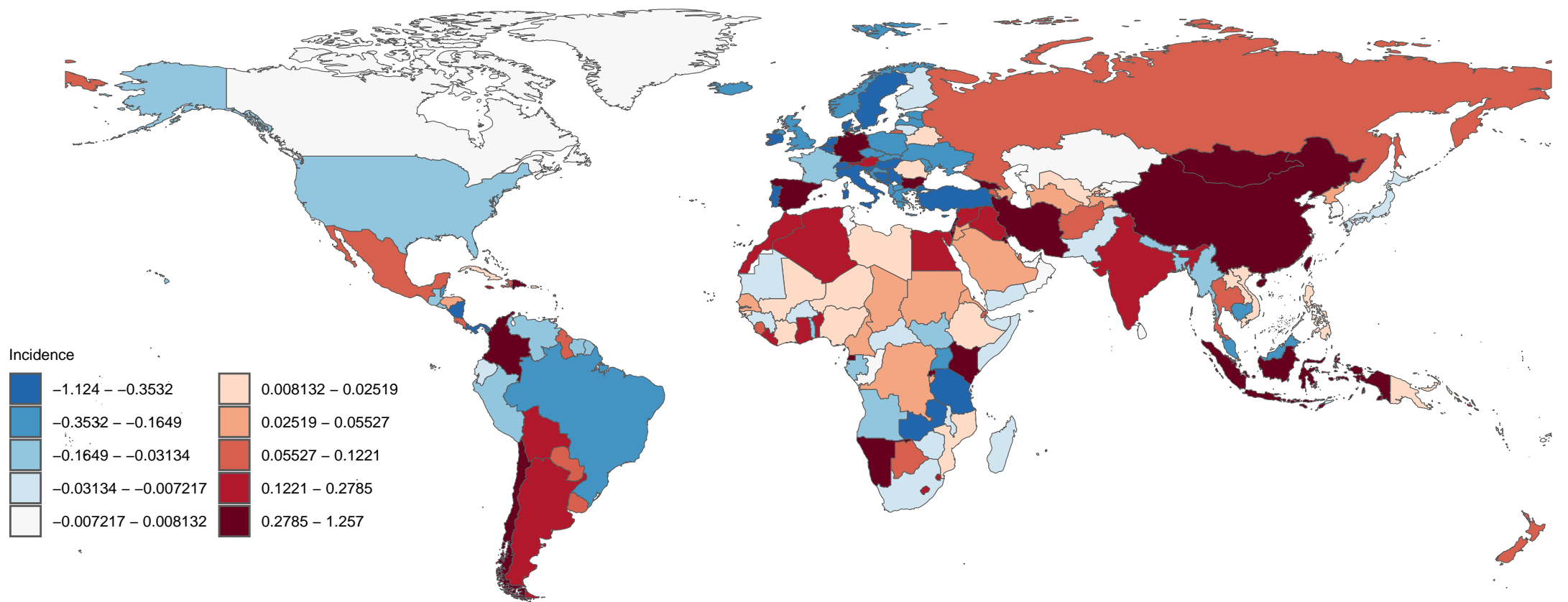

Supplement: Supplementary Figure 3 — Temporal trends and estimated annual percentage change (EAPC) of age-standardized incidence rate (ASIR) of Cannabis use disorders, from 1990 to 2021. [file DataSheet3.pdf]

DALYs (Disability-Adjusted Life Years)

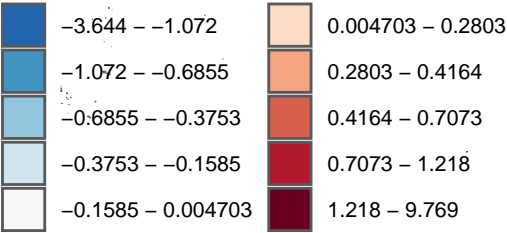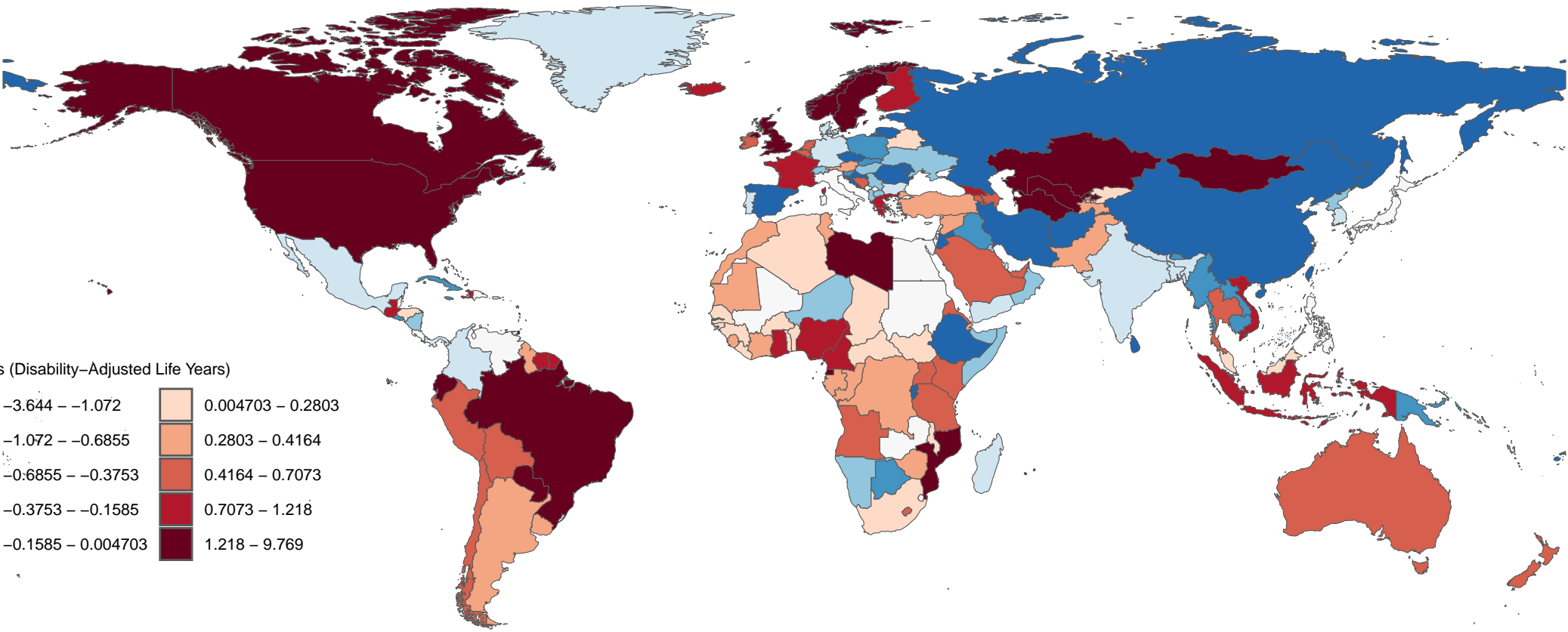

Supplement: Supplementary Figure 4 — Temporal trends and estimated annual percentage change (EAPC) of disability-adjusted life years (DALYs) of Cocaine use disorders, from 1990 to 2021. [file DataSheet4.pdf]

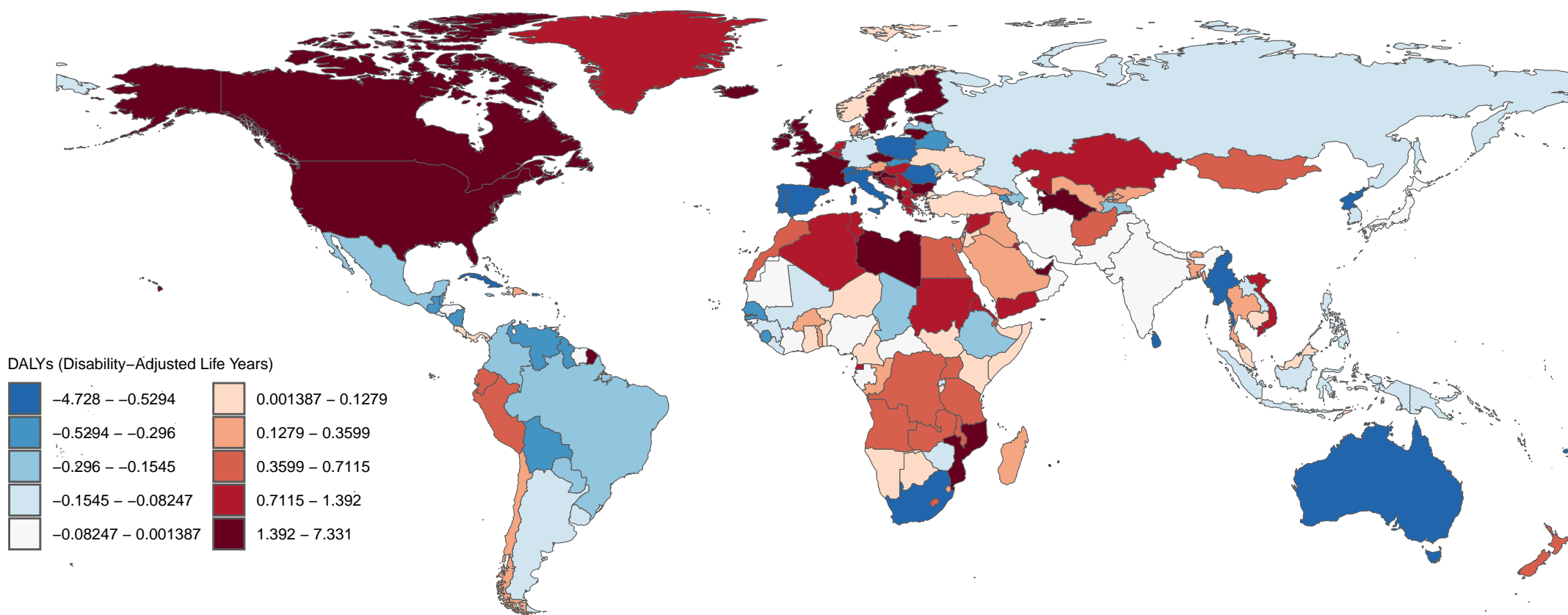

Supplement: Supplementary Figure 5 — Temporal trends and estimated annual percentage change (EAPC) of disability-adjusted life years (DALYs) of Opioid use disorders, from 1990 to 2021. [file DataSheet5.pdf]

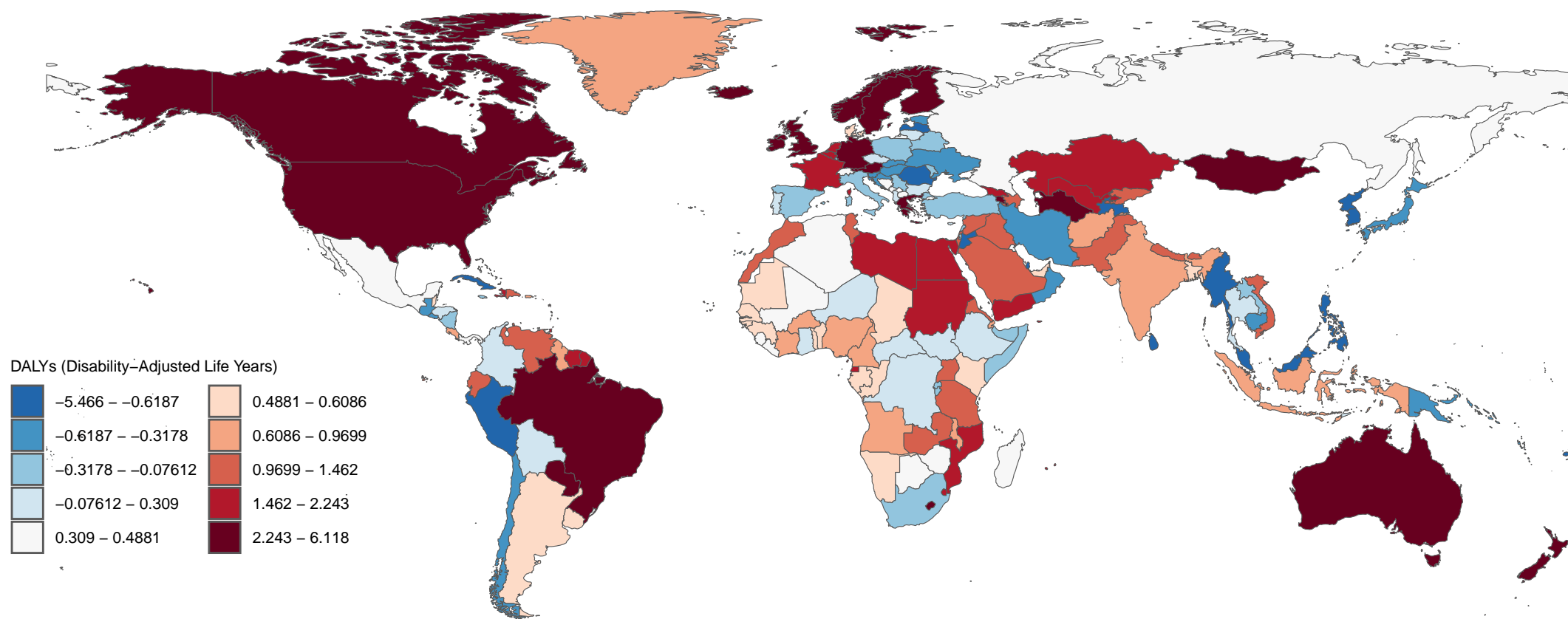

Supplement: Supplementary Figure 6 — Temporal trends and estimated annual percentage change (EAPC) of disability-adjusted life years (DALYs) of other use disorders, from 1990 to 2021. [file DataSheet6.pdf]
